# Covid-19 and Pregnancy: An Overview

**DOI:** 10.1055/s-0040-1713408

**Published:** 2020-06-19

**Authors:** Pedro Castro, Ana Paula Matos, Heron Werner, Flávia Paiva Lopes, Gabriele Tonni, Edward Araujo Júnior

**Affiliations:** 1 Department of Fetal Medicine, Clínica de Diagnóstico por Imagem, Rio de Janeiro, RJ, Brazil; 2 Department of Radiology, Universidade Federal do Rio de Janeiro, Rio de Janeiro, RJ, Brazil; 3 Prenatal Diagnostic Service, Department of Obstetrics and Gynecology, AUSL Reggio Emilia, Italy; 4 Department of Obstetrics, Escola Paulista de Medicina, Universidade Federal de São Paulo, São Paulo, SP, Brazil; 5 Medical Course, Universidade Municipal de São Caetano do Sul, São Caetano do Sul, SP, Brazil

**Keywords:** COVID-19, pregnancy, prenatal care, delivery, COVID-19, gravidez, pré-natal, parto

## Abstract

Since the World Health Organization (WHO) declared coronavirus infection (COVID-19) a Public Health Emergency of International Concern in January 2020, there have been many concerns about pregnant women and the possible effects of this emergency with catastrophic outcomes in many countries. Information on COVID-19 and pregnancy are scarce and spread throughout a few case series, with no more than 50 cases in total. The present review provides a brief analysis of COVID-19, pregnancy in the COVID-19 era, and the effects of COVID-19 on pregnancy.

## Resumo

Desde que a Organização Mundial da Saúde (OMS) declarou a infecção por coronavírus (COVID-19) uma emergência de saúde pública de interesse internacional em janeiro de 2020, houve muitas preocupações sobre mulheres grávidas e os possíveis efeitos dessa emergência com resultados catastróficos em muitos países. As informações sobre COVID-19 e gravidez são escassas e se espalham por algumas séries de casos, com não mais do que 50 casos no total. A presente revisão fornece uma breve análise da COVID-19, gravidez na era COVID-19 e os efeitos da COVID-19 na gravidez.

## Introduction

The coronavirus infection (COVID-19) is caused by the new virus labeled SARS-CoV-2. The first case was reported in Wuhan, China, in December 2019, and the illness rapidly spread throughout China and other countries. The infection typically presents as a fever and cough. Pneumonia is frequently observed in the diagnostic imaging tests of infected patients.^1^ The World Health Organization (WHO) estimates an overall mortality rate ranging from 3 to 4%, with a high rate of patients requiring admission to intensive care units (ICUs).^2^ ^3^

Pregnancy is a time of changes, both physical and psychological. Information about the impact of this infection on these changes is anxiously awaited by the medical community. There has been an unprecedently large number of medical publications. In the present article, we provide a brief analysis of COVID-19, pregnancy in the COVID-19 era, and the effects of COVID-19 on pregnancy, in addition to a critical analysis of COVID-19 in Italy.

## The Virus: History, Characteristics and Pathogenicity

SARS-CoV-2 belongs to the family Coronaviridae, order Nidovirales. Coronaviruses are enveloped, nonsegmented, positive-sense ribonucleic acid (RNA) viruses.^4^ The family Coronaviridae was first discovered in 1965 after growing in an embryonic tracheal culture obtained from an adult presenting with a common cold.^5^ After years of cataloging different viruses related to human and animal diseases such as gastroenteritis, hepatitis, and bronchitis, these viruses were called coronaviruses because of the crown-like appearance of their surface projections.^6^

During the 20^th^ century, studies inoculating the virus in voluntaries and epidemiological studies have reported an association between coronaviruses and respiratory diseases, but the virus was considered to have a low pathogenicity.^7^ ^8^ It is usually presented as a mild to moderate illness that was self-limited and lasted short periods of time.^9^

Four types of coronaviruses have been identified (α, β, gamma, and delta).^10^ They are classified according to their tropism and pathogenicity. The β viruses display great pathogenicity; they cause pneumonia and SARS and were responsible for the SARS-CoV and MERS-CoV outbreaks. Conversely, the α viruses usually present as mild to moderate upper respiratory tract infections.^11^ The recently identified SARS-CoV-2 presents a similarity between 20 and 60% to MERS-CoV and a similarity between 45 and 90% to SARS-CoV. However, it also presents great similarity with the genome of coronaviruses found in bats (96%).^12^ ^13^

The alveolar damage provoked by SARS-CoV and SARS-CoV-2 is likely caused by its reaction with the angiotensin-converting enzyme 2 (ACE 2), which is present mainly in type II pneumocytes.^14^ SARS-CoV-2 is hypothesized to have twice the affinity of SARS-CoV. The binding of ACE 2 to SARS-CoV or SARS-CoV-2 leads to its expression and subsequent alveolar damage. Angiotensin-converting enzyme 2 expression varies in different races and is more concentrated in men.^15^ ^16^ During inoculation, the virus crosses the mucosal membranes, mainly the nasopharynx and larynx, and reaches the pulmonary mucosa. In the lungs, the virus induces local inflammation and overtakes the systemic circulation, arriving to other organs that express ACE 2, such as the heart, lungs, and intestine.^17^ There is evidence of possible orofecal transmission.^18^ The virus presents a long life and stability in aerosols and on various surfaces.^19^

## Pregnancy and Respiratory Viruses

Pregnancy presents characteristics that make pregnant women more susceptible to respiratory pathogens and severe pneumonia. These changes include increased oxygen consumption, elevated diaphragm, and edema of the respiratory tract mucosa, which cause pregnant women to have an intolerance to hypoxia. This was noted during the H1N1 outbreak in 2009, in which pregnant women were four times more likely to be admitted to a hospital than the general population.^20^ Pneumonia is one of the more prevalent nonobstetric infections of pregnant women. It is the third most common indirect cause of maternal death and requires ventilatory support in 25% of cases.^21^ Despite the therapeutic options available for pulmonary infection, during pregnancy, the morbidity and mortality of viral infections are more severe than those from bacterial pneumonia.^22^ During the 1918–1919 outbreak, the maternal mortality rate reached 27%, and the risk increased proportionally with gestational age. When pneumonia was associated, the mortality rate reached 50%.^23^ Premature rupture of membranes, stillbirth, intrauterine growth restriction, and preterm birth are frequent complications of pulmonary infections.^24^

## Pregnancy and Covid-19

To determine the main symptoms and outcomes, a systematic review of the prenatal and perinatal effects of CoV infections on pregnancy was performed. A total of 538 articles were analyzed, and 27 were selected. When analyzing these data, it is important to note that the clinical manifestations were reported from women affected by severe forms of the disease, of whom 91.8% presented with pneumonia. Of these cases, 51.9% were COVID-19, 32.9% were SARS-CoV, and 15.2% were MERS-CoV. Of the pregnant women with CoV infection that evolved to pneumonia, 82.6% presented with fever, 57.1% presented with cough, and 27% presented with dyspnea. Lymphopenia was found in 79% of these women and elevated liver enzymes in 36.6%. In total, 34.1% were admitted to the ICU. Mechanical ventilation was required in 26.3% of the cases. When the most severe forms of CoV infections requiring ICU care were compared, COVID-19 presented better outcomes than SARS-CoV and MERS-CoV. A total of 9.3% of COVID-19 admissions were sent to the ICU, 5.4% required mechanical ventilation, and no maternal deaths were reported. For SARS-CoV and MERS-CoV, 44.6% and 53.3% of admissions were sent to the ICU, 40.9% and 40% required mechanical ventilation, and mortality rates of 28.6% and 25.8% were reported, respectively.^25^ These outcomes may be related to the vulnerability to respiratory pathogens and susceptibility to severe pneumonia that occur due to physiological adaptive changes during pregnancy (increased oxygen consumption, edema of respiratory tract mucosa, diaphragm elevation), leading the patient to have an increased intolerance to hypoxia. These modifications may be responsible for the increased vulnerability during the H1N1 influenza outbreak, in which pregnant women were four times more likely to be admitted to the hospital than the general population.^20^ Chest imaging may be required for the clinical evaluation and diagnosis of pregnant women with suspected COVID-19 infection. Chest X-ray examinations have little radiation exposure and can be helpful for diagnosis. Chest computed tomography (CT) has a high sensitivity for the diagnosis of COVID-19 and may be considered for diagnosis in epidemic areas.^26^ One study performed chest CT scans in 15 pregnant women infected with COVID-19. All presented with mild symptoms of the disease, and the gestational age ranged from 12 to 38 weeks. Ground-glass opacity was the most common early finding, and the lower pulmonary lobes were more affected.^27^ However, the effects of CoV infection on pregnancy have important prenatal and perinatal outcomes: miscarriage occurrence in 39.1% of infected pregnant women, premature rupture of membranes in 20.7%, preterm birth in 24.3% between 37 and 34 weeks, and preterm birth in 21.8% before 34 weeks. The effects of COVID-19 on fetal growth restriction and pre-eclampsia remain unknown. However, in SARS-CoV and MERS-CoV, fetal growth restriction occurred in 11.7% of pregnant women and pre-eclampsia in 16.2%. There were also high rates of cesarean section (83.9%). Moreover, perinatal deaths occurred in 11.1% of infected women, 34.6% of fetal distress, and 57.2% of admissions in neonatal ICU, and no neonatal asphyxia was reported. There was no evidence of vertical transmission in this series. When analyzed separately, the effects of the CoV infections presented very different results. There are no data about miscarriage in COVID-19 patients. MERS-CoV did not present miscarriages; however, SARS-CoV had a high miscarriage rate of 39%. MERS also did not increase premature rupture of the membranes and intrauterine growth restriction ratios, but premature rupture of the membranes occurred in 50% and 18.8% of pregnant women with SARS-CoV and COVID-19, respectively. Pre-eclampsia was absent in patients with SARS-CoV, but it occurred in 13.6% and 19% of pregnant women with COVID-19 and MERS, respectively. The preterm birth results deserve attention: in pregnant women with COVID-19, 41% of deliveries occurred before 37 weeks and 15% before 34 weeks. Preterm birth before 34 weeks occurred in 32.1% of pregnant women with MERS-CoV, before 34 weeks in 21.9%, and before 37 weeks in 15% of pregnant women with SARS-CoV.^25^ The prenatal care of women infected by COVID-19 or suspected/probable cases must be assessed every 2 to 4 weeks via ultrasound assessments of amniotic fluid volume and fetal growth evaluation, including umbilical artery Doppler when necessary.^28^ No evidence of vertical transmission was reported in a study involving nine patients. In this series, infection in a neonate was diagnosed; however, no evidence of vertical transmission was confirmed.^29^ This finding was also enforced in a study with 3 neonates and 230 children.^30^ Chinese guidelines recommend the isolation of COVID-19 positive neonates for 2 weeks if the mother is negative for COVID-19.^31^ Breastfeeding is not recommended for newborns from COVID-19 positive mothers according to Chinese Guidelines. The newborn must be isolated from the mother, based on two cases of newborn infections described.^32^

## Pregnancy in the Era of Covid-19

With the onset of the pandemic, investigations must be performed regarding the effects of the viremia during the first and second trimesters and the prediction of possible adverse outcomes. The higher rates of asymptomatic COVID-19 infections associated with the absence of recommendations for the routine detection or screening of COVID-19 during the first and second trimesters of the pregnancy may represent a challenge. Additionally, the effects of the stress and panic started by the onset of the global pandemic, in addition to the prolonged confinement, must be considered when assisting both noninfected and infected pregnant women. Therefore, all citizens, physicians, and particularly mothers-to-be are called to strictly follow designated guidelines. Thus far, it is clinically and socially useful to classify those who need a diagnostic test into probable, suspected, or confirmed SARS-CoV-2 (COVID-19) positive cases.^33^

A probable infected person is defined as one with a doubtful or inconclusive test to SARS-CoV-2 using RT-PCR to test for SARS-CoV-2. A suspected case is an individual with an acute respiratory infection with at least one of these symptoms (fever with a temperature >37.5°C, cough, and respiratory distress) of unexplained origin, or coming from a country where there is local transmission of the virus in the 14 days before the onset of symptoms, or a person with any type of acute respiratory infection that has been in contact with a probable or confirmed SARS-CoV-2 case in the 14 days before the onset of symptoms, or a person with a severe acute respiratory infection requiring hospitalization without another explanation for the clinical presentation. Last, a confirmed case is a positive result to a SARS-CoV-2 test performed by a registered laboratory independent of any clinical sign of the disease.^34^

Given that the current COVID-19 pandemic differs from the previous SARS-CoV outbreak of 2002 and the MERS-CoV outbreak of 2012 but has a higher human-to-human transmission rate, it is advisable to reduce access to hospitals or medical offices as much as possible unless respiratory symptoms arise: in these cases, nasopharyngeal swabs must be performed. If a mother-to-be accesses first aid facilities or requires admission to a hospital with symptoms of an acute respiratory infection, it must be considered a suspected case.^35^

A telephone triage is recommended if the mother is symptomatic or in self-isolation, and a clinical examination (where possible, and according to the degree of symptoms and fever) should be planned after several days. Mothers should be contacted by telephone for clinical follow-up with advice to alert the general practitioner if symptoms worsen. If the mother has a positive SARS-CoV-2 test, she will undergo a general clinical follow-up with ultrasound assessment of fetal growth every 4 to 6 weeks.^36^ Unless they are positive, the mothers should plan to attend appointments at an antenatal clinic at 36–37 weeks and at the hospital at 40 weeks.

## Covid-19 Effects in Pregnancy

Few case series on COVID-19 in pregnancy have been reported. The clinical series usually included less than 13 cases each, and all have reported on pregnancies primarily in the third trimester, demonstrating a lack of knowledge of the infection during the first and second trimesters. When these studies were analyzed together, the clinical manifestations were shown to usually develop after the 32^nd^ week. Given the few cases assessed during early gestational ages, clinical manifestations are usually observed close to late preterm and at delivery. Cesarean section was the preferred delivery mode for undescribed reasons, potentially because the patients were receiving oxygen therapy in most cases. However, the birthweight was usually normal in these studies.^37^ ^38^ ^39^

Fever is the main clinical manifestation and was present in > 78% of cases in the third trimester. Cough is the second manifestation to present in the infection. Sore throat was present in < 22% of cases, and dyspnea and diarrhea occurred in < 14%. It is important to note that a postpartum low-grade fever may indicate the need to test for COVID-19, as observed in a small series of five cases. All patients presented signs of pulmonary infection on CT and no other clinical manifestations.^40^

One case series of 13 pregnancies reported that 100% of cases were delivered via cesarean section. This report cites that 50% were emergency cesarean sections, mainly because of fetal distress, and there was one stillbirth. A total of 46% of 13 pregnancies had preterm birth. One woman developed severe pneumonia with multiple organ failure. Fetal distress was also reported in 22% of cases.^29^

The different types of reporting, in addition to different types of registering the findings and outcomes, as observed in the present analysis, leads to the necessity of a central, transparent, and accessible data center for COVID-19 cases. The majority of clinical manifestations were reported during the late third trimester; however, these conclusions may be biased because of the traditional conclusion of the cases in obstetrics, the maternal and newborn discharge. More studies are required to evaluate the effects of the infection during the early trimesters because no screening tests were suggested for the pregnant population to evaluate the effects in asymptomatic patients.^41^

## Antiviral Therapies in Pregnancy

In Wuhan, China, studies are currently being conducted for the medical treatment of the viral infection using two antiviral drugs, remdesivir and hydroxychloroquine (HCQ). Remdesivir has demonstrated antiviral activity in animal models of SARS-CoV and MERS-CoV and in certain tests.^42^ Hydroxychloroquine is an antirheumatic drug that has demonstrated an immunomodulatory capacity; it has been shown to prevent inflammation and organ damage and to reduce the proinflammatory signaling activation and cytokine production of IL-1, TNF, and type I interferons. In pregnant patients with autoimmune diseases, HCQ is strongly recommended for disease control and should be considered a potential therapeutic agent in cases of SARS-CoV-2 infections in pregnancy.^43^ Other medical treatment options are available even for pregnant patients. The association of lopinavir/ritonavir is not contraindicated in pregnancy, except for Kaletra oral solution, which is forbidden in pregnancy and in children < 14 years old. Darunavir/ritonavir should be evaluated case by case, and the use of darunavir/cobicistat is not recommended because there is evidence that pregnancy might reduce the pharmacological actions of active darunavir. Currently, there is a lack of data about possible teratogenic effects, effects on milk passage, or effects of remdesivir on neonates.^44^ ^45^

## Pregnancy and Health Care

A hydroalcoholic solution should be available for mothers coming to the waiting room, interpersonal distance of more than one meter must be maintained, and no accompanying persons are allowed unless the mother is not self-sufficient. In addition, the room should be ventilated every 10 minutes, and all surfaces and items should be disinfected.

If a symptomatic mother is present in the waiting room, she must wear a surgical mask and be assessed as soon as possible. Moreover, ventilating and disinfecting the waiting room is mandatory. If the mother is asymptomatic, the use of a surgical mask is considered unnecessary unless respiratory symptoms are present in the health caregivers; however, proper hand hygiene must be observed before and after each individual examination. If the mother is symptomatic (see definition above), she will be invited to wear a surgical mask while the health caregivers are called to wear a mask, glasses, gloves, and disposable gowns. The surface must be cleaned with disinfectant (alcohol solution at least 75%), and the room should be ventilated. In addition, nasopharyngeal swabs must be taken together with complete isolation in a dedicated room, and the Office of Public Hygiene should be informed. If the general condition of the mother is stable, she will be advised to observe a period of isolation of 14 days, and in those testing SARS-CoV-2 positive, a clinical and ultrasound follow-up will be planned every 4 to 6 weeks onwards. If her general condition is unstable or poor, the mother will be admitted to a referral tertiary care center.

Different management strategies should be arranged to assist mothers in the delivery room. For asymptomatic mothers, the general rules described above should be followed, and depending upon availability, health caregivers should wear surgical masks. As noted above, only one accompanying person is allowed in the delivery room unless he/she has tested positive for SARS-CoV-2.

**Fig. 1:**
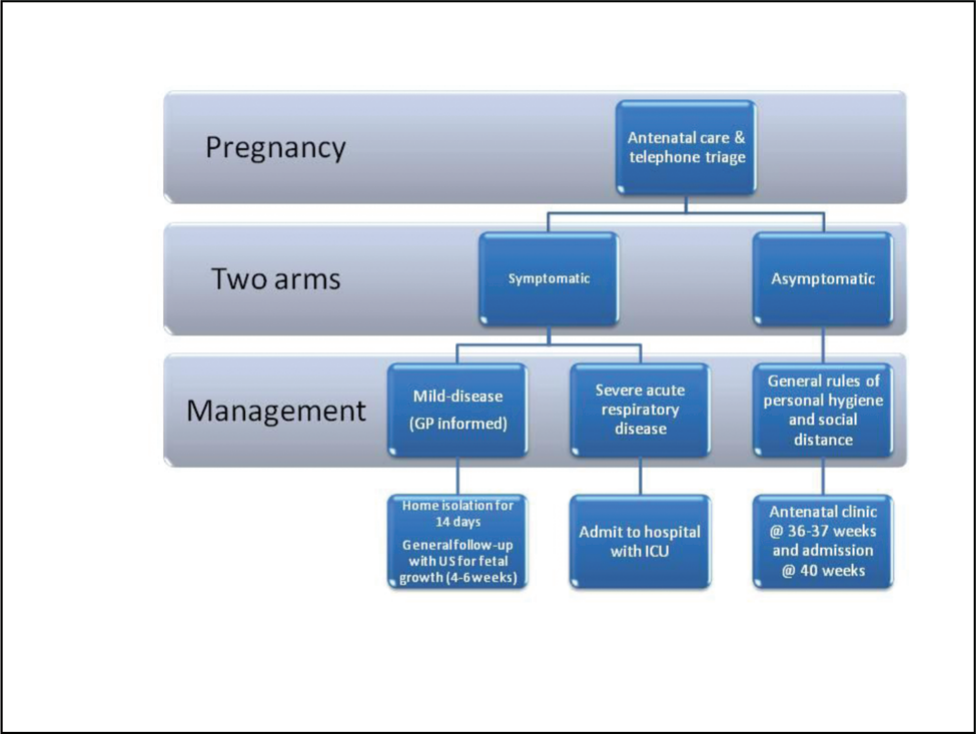
Flowchart of the antenatal prenatal care.

**Fig. 2:**
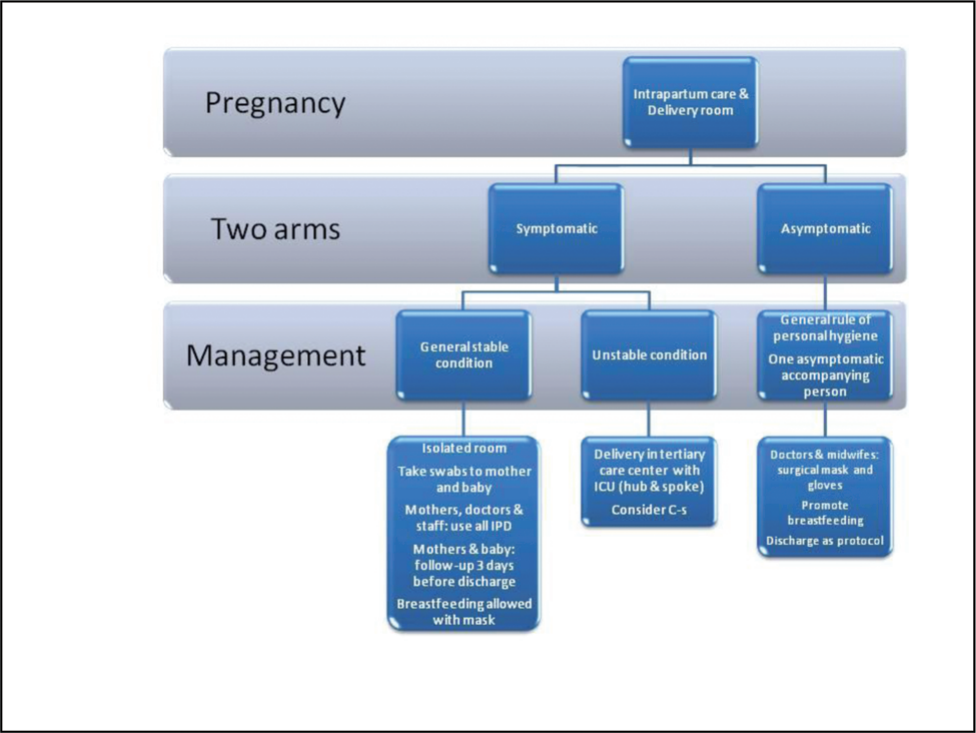
Flowchart of the delivery room.

If the mother is symptomatic with a general stable condition, the use of all individual protection devices is mandatory for both mothers and doctors/midwifes/nurses, and maternal and neonatal swabs must be taken. Both the mother and baby must be kept in an isolated room, and secondary to the swab results, they must be followed up for 3 days before hospital discharge. If the mother is symptomatic but in an unstable condition, referral to a tertiary care unit with a resuscitation facility should be arranged. Acute infection with COVID-19 does not represent per se an indication for cesarean section during intrapartum or a contraindication for breastfeeding given that the mother observes proper hand washing and hygiene every time before and after breastfeeding and will continue to wear a surgical mask.^46^ Only one accompanying person is allowed: if respiratory symptoms are present, all the aforementioned individual protection methods will be strictly followed. The admittance of a suspected or known SARS-CoV-2 positive individual is forbidden. All recommendations for protection will be strictly undertaken. Separating mothers and their newborns should be avoided, and breastfeeding should be encouraged according to the will and right of the mother.^47^ There is supporting evidence of a lack of vertical transmission,^48^ the virus has not been found in the amniotic fluid, cord blood, or maternal milk,^49^ ^50^ and there are no registered cases of maternal death.^51^ A possible explanation might be the absence of the ACE receptor at the fetal-maternal interface, which was the opposition of that for the human AXL protein in the Zika virus infection.^52^ ^53^ ^54^ Figs. 1 and 2 show the flowcharts of the antenatal prenatal care and delivery room, respectively, which were in part developed by the Instituto Superiore Sanità (ISS, www.iss.it) and Emilia-Romagna Region (www.salute.regione.emilia-romagna.it) (Tables 1 and 2).

**Table 1:** Maternal and perinatal outcomes of pregnant women with Covid-19

	Cases	Maternal Age (years old)	Gestational age at clinical manifestation (weeks)	Gestational age at delivery	Methods of Diagnosis	Delivery Mode	Birthweight (grams)
Chen et al^29^	9	28 (26–40)	37^+2^ (36–39^+4^)	37^+2^	qRT-PCR	9 CS	2970 (1880–3820)
Zhu et al^53^	7	30 (25–34)	34^+5^ (30^+4^-38^+4^)	34^+6^	CT	6 CS 1 VD	2300 (1520–3800)
Liu et al^27^ *	11	32 (23–40)	2–19 days before admission at 32w (12–38)	NA	CT	10 CS 1 VD	NA
Yu et al^37^	7	32 (29–34)	5 days (2–9) before admission at 39w (37–41)	39^+2^(37–41^+5^)	qRT-PCR and CT	7 CS	3250 (3000–3500)
Chen et al^40^	5	29 (25–31)	39^+1^ (38^+6^-40^+3^)	39^+1^ (38^+6^-40^+4^)	qRT-PCR	2 CS 3 VD	3700 (3235–4050)
Lee et al^38^	1	28	36^+2^	37^+6^	qRT-PCR	CD	3130
Liu et al^54^	13	22–36	2 days before 28 weeks and 11 days on third trimester	NA	qRT-PCR	10 CD (100%)	NA

Abbreviations: CS, cesarean section; CT, computed tomography; NA, not available; VD, vaginal delivery; w, weeks.

* data including three puerperal patients.

**Table 2:** Main clinical manifestation of pregnant women with Covid-19

	Cases	Fever	Cough	Sore throat	Diarrhea	Dyspnea
Chen et al^29^	9	78%	44%	22%	11%	11%
Zhu et al^53^	7	100%	42%	14%	14%	NA
Liu et al^27^ *	11	86%	59%	6%	6%	6%
Yu et al^37^	7	85%	14%	NA	14%	14%
Chen et al^40^	5	0%	0%	NA	NA	0%
Lee et al^38^	1	Present	Present	Present	NA	NA
Liu et al^54^	13	77%	NA	NA	NA	23%

Abbreviations: NA, Not available.

* data including three puerperal patients.

Now is a time of new perspectives regarding dealing with infections of pandemic proportions. Much will be learned from this stress test on the health care systems and how they cope, and the successes and failures of recommendations and guidelines from health organizations and their timing will be analyzed. However, much can and has already been done to create solutions for clear communication between medical scientists.^55^ The cases reported mainly focused on patients with evident clinical manifestations, in whom the disease can progress quickly and become severe in a few hours. However, there is evidence showing that pulmonary imaging findings may be evident in very early stages of COVID-19 infection. These data can change the course of the traditional care of respiratory infections, when imaging is reserved for worsening clinical conditions.^56^ Additionally, the consequences of a period of intense stress and anxiety during pregnancy, as spontaneous preterm labor and psychiatric disease on the siblings are already known, and special attention must be reserved for those pregnant, noninfected women, in this time of an overloaded health care system and exhausted health care workers.^57^
